# Investigation of the association between habitual dietary FODMAP intake, metabolic parameters, glycemic status, and anthropometric features among apparently healthy overweight and obese individuals

**DOI:** 10.1186/s12902-023-01458-4

**Published:** 2023-09-26

**Authors:** Reyhaneh Mokhtari Hemami, Amir Shakarami, Abnoos Mokhtari Ardekani, Sara Aghaii, Dorna Makarem, Negin Nikrad, Mahdieh Abbasalizad Farhangi, Mohammad Sadegh Pour Abbasi

**Affiliations:** 1 Tabriz Health Services Management Research Center, Tabriz University of Medical Sciences, Tabriz, Iran; 2 Department of Cardiovascular Medicine, Lorestan University of Medical Sciences, Khorramabad, Iran; 3 Endocrinology and Metabolism Research Center, Institute of Basic and Clinical Physiology Science, & Physiology Research Center, Kerman University of Medical Sciences, Kerman, Iran; 4 Faculty of Medical Sciences, Tehran University of Medical Sciences, Tehran, Iran; 5 Escuela Tecnica Superior de Ingenieros de Telecomunicacion Politecnica de Madrid, Madrid, Spain; 6 Department of Community Nutrition, Faculty of Nutrition, Tabriz University of Medical Sciences, Tabriz, Iran; 7 Department of Cardiovascular Surgery, Kashan University of Medical Sciences and Health Services, Kashan, Iran

**Keywords:** FODMAP diet, Metabolic syndrome, Dyslipidemia, Obesity

## Abstract

**Background:**

The predisposition of humans to metabolic syndrome is affected by many factors, including diet and lifestyle. Fermentable oligosaccharides, disaccharides, monosaccharides, and polyols (FODMAPs) are a set of carbohydrates that are fermented by gut microbiota. In animal studies, supplementation with FODMAP-rich diets as prebiotics can alter body composition and gut microbiota. This study evaluates any relationship between FODMAP and metabolic syndrome risk factors among adults with metabolic syndrome in Iran.

**Methods:**

This cross-sectional study is based on sociodemographic information from 347 overweight and obese participants selected from outpatient clinics through public declaration. Participants body composition and anthropometric measures were also determined. A validated Food Frequency Questionnaire (FFQ) with 168 questions was used to collect dietary data. Biochemical parameters, including serum total cholesterol (TC), triglycerides (TG), high-density lipoprotein cholesterol (HDL-C), fasting serum glucose (FSG), and insulin levels, were determined by enzymatic methods. In addition, the Homeostasis Model Assessment of Insulin Resistance (HOMA-IR) and Quantitative Insulin Sensitivity Check Index (QUICKI) were calculated.

**Results:**

In moderate FODMAP and low FODMAP groups, lower waist-to-hip ratio (WHR) and higher fat-free mass (FFM) were achieved in higher tertiles. In high FODMAP groups, higher systolic blood pressure (SBP) was shown in the higher tertile (*P* < 0.05). Higher insulin, HOMA-IR, and lower QUICKI in the second tertile of the high FODMAP group were also observed.

**Conclusion:**

Findings of this study highlight the potential role of FODMAP in managing metabolic syndrome and open a new field of research.

## Background

The predisposition of humans to metabolic diseases is affected by many factors, including diet and lifestyle. Metabolic syndrome (MetS) is a cluster of conditions that occur together, increasing the risk of heart disease, cerebral vascular accident (CVA), and type 2 diabetes. These conditions include increased blood pressure, high fasting blood sugar, excess body fat, and elevated serum cholesterol or triglyceride [1, 2]. Any metabolic syndrome elements increase the risk of developing cardiovascular disease (CVD), type 2 diabetes mellitus, and CVA [3]. Obesity is a newly found factor associated with the high prevalence of metabolic syndrome [4, 5]. Although there is great genetic background for developing overweight and obesity [6], environmental factors are thought to be responsible for the recent dramatic increase in the prevalence of obesity [7]. The difference between the amount of energy consumed and the amount of energy expended leads to the storage of excess energy as fat, resulting in obesity. Recent evidence has found the significant role of gut microbiota in obesity [8]. As a result, therapeutic approaches based on manipulating gut microbiota, such as probiotics and prebiotics, are developed for treating obesity and metabolic syndrome [9].

Fermentable oligosaccharides, disaccharides, monosaccharides, and polyols (FODMAPs) are short-chain carbohydrates metabolized and fermented by gut microbiota [10]. Low FODMAP diets were the first treatment to reduce irritable bowel syndrome symptoms [11, 12]. A low FODMAP carbohydrate diet promotes Bacteroides while decreasing Bifidobacterium [13–15] and Akkermansia muciniphila, which have beneficial metabolic effects. The balance of deconjugated secondary bile acids [13], short-chain fatty acid (SCFA) [14], lipopolysaccharide (LPS) [8], and incretin secretion [15–17] are altered through the generation of active metabolites by these microbiotas during fermentation of FODMAP carbohydrates, which can change the metabolism of glucose and lipid. In one animal study, supplementation with FODMAP-rich diets as prebiotics altered body composition and gut microbiota [18]. The study also has shown that a high-fat diet rich in fructooligosaccharides (FOS) decreases mass and adiposity in rats [18]. Other studies on mice showed that a high-fat diet mixed with galactooligosaccharides (GOS) supplementation decreases LDL-cholesterol, elevates Bifidobacterium level, and reduces Clostridium [19]. In another rodent study, GOS increased the incretin hormones, glucagon-like peptide-1 (GLP-1) and peptide YY (PYY), and abundance of health-promoting Bifidobacterium [20]. In 5 randomized clinical trials (RCT) involving 44 overweight/obese subjects with pre-diabetes, adding 15 g of GOS daily to a regular diet increased Bifidobacterium by 5-times but insulin sensitivity, SCFA, and LPS were not changed [21–23]. Therefore, due to a limited understanding of the relationship between FODMAP and MetS, much more investigation is needed to evaluate the association between FODMAP and the risk factors of MetS. Accordingly, this study aims to evaluate the relationship between FODMAP and metabolic syndrome risk factors among adults with metabolic syndrome in Iran.

## Materials and methods

This cross-sectional study included 347 overweight and obese participants in Tabriz and Tehran, Iran. The study protocol was approved and registered by the ethics committee of Tabriz University of Medical Sciences (registration code: IR.TBZMED.REC.1402.330).

### Inclusion–exclusion criteria

Two recent projects were previously conducted in the Tabriz and Tehran cities of Iran [24, 25]. Individuals were selected from outpatient clinics through public declaration and the dissemination of posters. The inclusion criteria for this study were individuals aged between 20 to 50 and a BMI of 25 kg/m^2^ and more. Individuals with specific conditions, including pregnancy, breastfeeding, menopause, recent bariatric surgery, a history of cardiovascular disease (CVD), cancer, hepatic or renal disease, diabetes mellitus, and taking any drugs and medications that affect weight, were excluded from the study. Participants who had been on a weight-loss regimen or taking supplements for at least three months before participating were excluded from the study.

### Demographics and anthropometric evaluations

We gathered sociodemographic information, including age, gender, smoking status, educational level, marital status, employment, past medical history, and family size, by asking the participants to fulfill a questionnaire. The socioeconomic status (SES) score was then computed. Then, we categorized participants’ education level using ordered categorical variables: illiterate: 0, less than a diploma: 1, diploma and associate degree: 2, bachelor’s degree: 3, master’s degree: 4, and higher: 5. The occupational status was also recorded similarly: housewife: 1, a worker: 2, student: 3, freelancers:4 and more: 5 for females; And without a job: 1, rancher, farmer, and worker: 2, extras: 3, employee: 4, and independently employed: 5 for men. Additionally, individuals were assigned scores of 1, 2, or 3 to indicate whether they had a family size of 3, 4–5, or 6, respectively. They also received a score of 1 if they did not own a house and a score of 2 if they did. The body composition was determined using bioelectrical impedance analysis (BIA) (Tanita, BC-418 MA, Tokyo, Japan). A wall-mounted stadiometer and a Seca scale (Seca Co., Hamburg, Germany) were used to measure height and weight to the nearest 0.5 cm and 0.1 kg, respectively. The hip circumference (HC) was measured across the broadest part of the buttocks just upon the greater trochanters. The waist circumference (WC) was measured using tape to the nearest 0.1 cm at the midpoint of the lowest costal border and the iliac crest. We also calculated the waist-to-hip ratio (WHR) and the body mass index (BMI). Using a standard, calibrated mercury sphygmomanometer (Riester, Diplomat 1002, Jungingen, Germany), blood pressure was measured twice in the same arm after at least 15 min of rest. The mean of the two measurements was used for analysis. The US National Cholesterol Education Program Adult Treatment Panel III (NCEP-ATP III) criteria defined MetS [26]. The short form of the International Physical Activity Questionnaire (IPAQ) was used to measure the physical activity levels among participants [27–29].

### Dietary assessment and its reliability and validity

A validated semi-quantitative Food Frequency Questionnaire (FFQ) with 168 questions was used to collect dietary data for the Iranian population [30]. Participants kept diaries detailing how often and how much of each food item they consumed each day, week, month, and year. The amount of food consumed was converted to grams per day using the standard common portion size, cooking yield factors, and edible portions of foods found in the Iranian household measures manual [31].

The NUTRITIONIST IV software (N Squared Computing, California, USA) was utilized to analyze daily dietary intakes. The validity and dependability of the FFQ used in this study were previously evaluated [30]. Based on the estimated validity coefficients, reasonable relative validity was obtained. Men and women had nearly identical correlation coefficient values for various nutrients. The food groups were specified as follows: Whole grains, refined grains, potatoes, dairy products, vegetables, fruits, legumes, meats, nuts and seeds, solid fat, liquid oil, tea and coffee, salty snacks, simple sugars, honey and jam, soft drinks, and desserts and snacks. The frequency with which people added salt or salty sauce to food while it was being prepared or cooked, before or during eating, and the frequency of consuming processed foods with a high salt content was used to assess dietary salt consumption [32].

The foods were categorized based on their FODMAP content into high, moderate, and low FODMAP groups using the classification system provided by the Monash University Android app [33]. The Monash Uni low FODMAP diet was developed by nutritionists who coined the term FODMAP and is regularly updated and accessible globally [34]. Iranian foods were adopted with the list of high FODMAP (E.g., wheat, garlic, onion, fruit, vegetables, legumes and pulses, sweeteners, and other grains), moderate FODMAP (E.g., avocado, sweet potato, broccoli, cabbage, canned pumpkin) and low FODMAP (E.g., eggs and meat, almond milk, grains like rice, quinoa and oats, vegetables like eggplant, potatoes, tomatoes, cucumbers and zucchini, fruits such as grapes, oranges, strawberries, blueberries, and pineapple) foods. Then the consumed amounts of low, moderate, and high FODMAP foods for each participant were calculated using the FFQ described before. In order to calculate the gram of high, moderate, and low FODMAP food intake, we used a semi-quantitative food frequency questionnaire in which every food item each participant consumed was converted into grams. The sum of all foods with high, moderate, or low FODMAP content that each participant consumed was calculated in grams separately. Then, these final values were categorized into tertiles. Then we categorized it into three tertiles (based on the amount of consumption). Higher tertiles denote higher consumption of dietary FODMAP.

### Biochemical evaluation

A total of 10 ml of fasting venous blood were obtained from all participants for the biochemical analysis. A commercial kit (Pars Azmoon, Tehran, Iran) was utilized to determine total serum cholesterol (TC), triglyceride (TG), high-density lipoprotein cholesterol (HDL-C), and fasting blood glucose (FBG). Plasma and serum samples were divided by centrifugation at 4,500 rpm for ten minutes at four degrees Celsius. Aliquots were frozen at 70 degrees Celsius prior to the analysis. Moreover, to calculate the portion of low-density lipoprotein cholesterol (LDL-C), the Friedewald equation was applied [35]. Enzyme-linked immunosorbent assay (ELISA) kits (Bioassay Technology Laboratory, Shanghai Korean Biotech, Shanghai City, China) were utilized to determine insulin levels in the blood. The Quantitative Insulin Sensitivity Check Index (QUICKI) and the Homeostasis Model Assessment of Insulin Resistance (HOMA-IR) were calculated by dividing fasting insulin (IU/ml) by 22.5 fasting glucose (mmol/l). 1/[log insulin (U/mL) + log glucose (mmol/L) during fasting].

### Statistical analysis

The SPSS (IBM SPSS version 26.0) software was used to analyze the data at a significance level of 0.05. Categorical variables and continuous variables were described as frequency (percentage) and mean [standard deviation (SD)], respectively. Analysis of variance (ANOVA) was utilized to evaluate the association between low, moderate, and high FODMAP foods and metabolic syndrome. Analysis of covariance (ANCOVA) was used to control the effect of confounding variables (including age, sex, BMI, and total energy intake) on the association of low, moderate, and high FODMAP foods and metabolic syndrome. Multinomial logistic regression was used to estimate odds ratios (ORs) and 95% confidence intervals (CIs) for the presence of cardiometabolic risk factors across the FODMAP tertiles in two multivariable-adjusted models. The risk was described in three models (Model I: crude, Model II: adjusted for age and sex, Model III: adjusted for age, BMI, sex, SES, and energy intake). The G-power software was utilized to determine the minimum sample size required for the study, considering a correlation coefficient (r) of 0.25, a significance level of 0.05, and a power of 80%, which resulted in a prediction of 315 participants. However, based on previous studies, considering a 10% drop-out [36, 37]. The sample size was calculated with α = 0.05 and β = 0.2. Therefore, the power was 80%. According to the power of 80%, categorizing of the high, moderate, and low FODMAP groups into tertiles was the best choice to avoid false positives due to multiple comparisons and false negatives due to inadequate power [38, 39]. The final sample size for the study was 347 individuals, 58.2% male and 41.8% female [40]. The sampling was performed in three months.

## Results

The general demographic and anthropometric features of study participants are represented in Table 1. As shown, there was no significant difference in general characteristics and anthropometric variables among different tertiles of high FODMAP. However, in moderate and low FODMAP groups, lower WHR and higher FFM were achieved in higher tertiles (*P* < 0.05). Also, those at the higher tertile of the moderate FODMAP group had significantly higher BMR than other tertiles (*P* = 0.04). Also, as shown in Table 1, male subjects consumed higher amounts of moderate and low FODMAP foods (*P* < 0.001 and 0.01, respectively). Table 2 compares biochemical variables in high, moderate, and low FODMAP groups in crude and energy, age, gender, physical activity, and BMI-adjusted models. No significant association was observed in high, moderate, and low FODMAP groups. Tables 3, 4, and 5 show the odds of biochemical variables in second and third tertiles versus first in the high, moderate, and low FODMAP groups of food items. In the high FODMAP group (Table 3), individuals in the third tertile of the high FODMAP group were more likely to have higher SBP and DBP than those in the first tertile (*P* < 0.05) in crude and age, sex-adjusted models. Also, higher serum insulin levels, HOMA-IR, and lower QUICKI in the second tertile versus the first tertile of the high FODMAP group were observed. In the moderate FODMAP group (Table 4), those at the second tertile were less likely to have higher SBP than the first tertile (OR = 0.954; CI = 0.919–0.991; *P* = 0.01) in the fully-adjusted model. No significant association was observed for the low FODMAP group (Table 5) for biochemical variables in multinomial logistic regression models. Tables 6 and 7 compare dietary macronutrients, and some of the micronutrients and food groups across different tertiles of dietary low, moderate, and high FODMAP groups. As expected, there was an increase in almost all of the food ingredients and food groups in different tertiles of dietary low, moderate, and high FODMAP groups.

**Table 1:** Anthropometric measurements of study participants across different tertiles of High FODMAP, Moderate FODMAP and Low FODMAP intake

**Variables**	**High FODMAP (0.02–1.22g/ kcal)**				**Moderate FODMAP** **(0.03–0.39 g/ kcal)**				**Low FODMAP** **(0.13–2.96 g/ kcal)**			
	**1**^**st**^** tertile (*n* = 113)**	**2**^**nd**^** tertile (*n* = 113)**	**3**^**rd**^** tertile (*n* = 113)**	*P	**1**^**st**^** tertile (*n* = 113)**	**2**^**nd**^** tertile (*n* = 113)**	**3**^**rd**^** tertile (*n* = 113)**	*P	**1**^**st**^** tertile* (n* = 113)**	**2**^**nd**^** tertile (*n* = 113)**	**3**^**rd**^** tertile (*n* = 113)**	*P
**Age (year)**	39.30 (7.93)	40.72 (9.39)	41.84 (10.01)	0.11	41.30 (9.40)	40.71 (9.42)	39.92 (8.72)	0.52	40.01 (10.04)	41.20 (8.14)	40.71 (9.29)	0.62
**SES**	9.96 (2.62)	10.00 (2.54)	10.02 (2.20)	0.09	9.63 (2.70)	10.56 (2.18)	9.83 (2.50)	0.11	9.89 (2.73)	9.51 (2.26)	10.41(2.46)	0.12
**Education (≤ 12 y)**	73 (92.4)	59 (93.7)	41 (93.2)	0.75**	59 (90.8)	49 (96.1)	65 (91.6)	0.66**	56 (94.9)	54 (90.0)	63 (92.6)	0.87**
**Marital status (% Single)**	15 (13.3)	13 (11.5)	17 (15.0)	0.72**	10 (8.8)	18 (15.8)	17 (15.0)	0.43**	12 (10.6)	13 (11.4)	20 (17.7)	0.15**
**Gender (%Male)**	66 (58.4)	70 (61.9)	60 (53.1)	0.39**	52 (46.0)	72 (63.2)	72 (63.7)	< 0.001**	55 (48.7)	64 (56.1)	77 (68.1)	0.01**
**Weight (kg)**	92.59 (13.31)	92.72 (14.28)	90.89 (15.86)	0.57	90.10 (15.76)	91.85 (14.29)	94.34 (13.12)	0.08	90.67 (15.60)	92.76 (14.43)	92.86 (13.40)	0.43
**Height (cm)**	167.74 (9.64)	168.57 (10.20)	167.66 (9.80)	0.74	166.04 (10.41)	169.15 (8.96)	168.66 (9.99)	0.03	167.1 (10.12)	168.25 (10.32)	168.45 (9.17)	0.57
**BMI (kg/m**^**2**^**)**	32.97 (4.50)	32.57 (4.48)	32.32 (5.42)	0.59	32.72 (5.32)	32.007 (4.24)	33.24 (4.85)	0.15	32.43 (4.83)	32.86 (5.26)	32.66 (4.40)	0.79
**WC (cm)**	106.95 (9.69)	106.77 (9.17)	106.32 (10.13)	0.88	105.35 (10.54)	106.61 (8.69)	108.17 (9.50)	0.08	105.33 (9.38)	107.03 (9.92)	107.77 (9.55)	0.15
**HC (cm)**	115.27 (9.16)	115.30 (9.16)	113.96 (9.44)	0.52	115.69 (9.39)	113.36 (8.14)	115.65 (9.99)	0.12	114.93 (8.84)	115.42 (10.56)	114.30 (8.16)	0.69
**WHR**	0.93 (0.08)	0.93 (0.07)	0.93 (0.07)	0.95	0.91 (0.08)	0.94 (0.07)	0.94 (0.07)	**0.01**	0.91 (0.082)	0.93 (0.081)	0.94 (0.061)	**0.03**
**FM (kg)**	33.44 (8.24)	32.57 (8.94)	35.97 (10.53)	0.15	35.33 (9.88)	31.47 (6.98)	34.09 (9.54)	0.74	35.34 (8.94)	34.41 (9.86)	31.92 (8.40)	0.08
**FFM (kg)**	61.98 (12.95)	63.78 (11.78)	60.54 (12.14)	0.37	58.19 (12.89)	65.35 (11.89)	63.73 (11.32)	** < 0.001**	58.57 (12.50)	63.28 (11.64)	64.59 (12.26)	**0.01**
**BMR (kcal)**	7887.26 (1509.45)	8088.85 (1377.80)	7601.78 (1856.96)	0.28	7460.01 (1521.28)	8137.96 (1799.19)	8014.65 (1517.24)	**0.04**	7518.41 (1477.33)	7943.76 (1581.95)	8077.37 (1738.17)	0.13

All data are mean (± SD) except marital status and gender, that is presented as the number and percent of single and males respectively in each group

*BMI* Body mass index, *WC* Waist Circumference, *FM* Fat Mass, *FFM* Fat Free Mass, *WHR* Waist-to-hip ratio, *BMR* Basal Metabolic Rate, *SES* Socio-economic status

^*^P values derived from One-Way ANOVA

^**^P values derived from chi-squared test

**Table 2:** Biochemical parameters of study participants across different tertiles of High FODMAP, Moderate FODMAP and Low FODMAP intake

**Variables**	**High FODMAP** **(**0.02–1.22g/ kcal)					**Moderate FODMAP** **(**0.03–0.39 g/ kcal**)**					**Low FODMAP** **(**0.13–2.96 g/ kcal**)**				
	**1**^**st**^** tertile (*n* = 113)**	**2**^**nd**^** tertile (*n* = 113)**	**3**^**rd**^** tertile (*n* = 113)**	*P	**P	**1**^**st**^** Tertile(*n* = 113)**	**2**^**nd**^** tertile (*n* = 113)**	**3**^**rd**^** tertile (*n* = 113)**	*P	**P	**1**^**st**^** Tertile (*n* = 113)**	**2**^**nd**^** tertile (*n* = 113)**	**3**^**rd**^** tertile (*n* = 113)**	*P	**P
**SBP (mmHg)**	119.84 (14.29)	122.18 (18.23)	126.29 (15.91)	**0.01**	0.46	123.59 (14.60)	123.03 (14.65)	121.92 (19.67)	0.742	0.13	121.69 (17.77)	123.93 (14.56)	122.91 (16.90)	0.59	0.33
**DBP (mmHg)**	80.19 (10.35)	81.07 (13.23)	83.40 (10.70)	0.09	0.61	82.30 (11.07)	82.03 (10.47)	80.67 (13.43)	0.535	0.60	80.69 (13.00)	83.28 (11.16)	81.01 (10.80)	0.19	0.08
**TC (mg/dL)**	188.12 (35.02)	196.05 (35.69)	190.92 (39.08)	0.25	0.81	192.14 (39.06)	191.33 (36.28)	191.92 (34.89)	0.985	0.36	193.07 (37.36)	193.18 (39.47)	189.14 (33.04)	0.64	0.65
**TG (mg/dL)**	141.75 (81.28)	146.31 (90.17)	164.61 (105.93)	0.15	0.79	139.48 (68.29)	159.57 (112.84)	153.73 (92.28)	0.25	0.10	147.90 (100.26)	165.35 (108.85)	139.55 (62.69)	0.10	0.24
**HDL-C (mg/dL)**	45.25 (8.94)	45.88 (9.36)	44.70 (9.89)	0.64	0.46	46.33 (9.66)	44.13 (9.26)	45.33 (9.18)	0.20	0.11	45.47 (9.22)	45.19 (10.11)	45.12 (8.85)	0.95	0.75
**LDL-C (mg/dL)**	120.46 (31.48)	126.65 (32.15)	123.55 (32.14)	0.34	0.85	124.99 (33.21**)**	123.67 (30.15)	122.26 (32.56)	0.81	0.70	126.48 (33.20)	124.18 (33.31)	120.26 (29.02)	0.33	0.84
**Glucose (mg/dL)**	91.95 (13.25)	92.89 (16.84)	93.14 (25.80)	0.89	0.33	92.57 (14.30)	89.80 (13.32)	95.90 (27.04)	0.05	0.14	91.14 (12.24)	92.80 (17.86)	94.29 (25.62)	0.47	0.78
**Insulin (µIU/mL)**	16.35 (16.50)	16.25 (9.75)	15.37 (13.07)	0.88	0.12	16.86 (11.33)	13.88 (7.17)	17.04 (18.58)	0.26	0.70	16.07 (10.50)	18.43 (18.86)	13.62 (8.00)	0.06	0.50
**HOMA-IR**	3.72 (3.59)	3.82 (2.51)	3.64 (3.49)	0.94	0.14	3.92 (2.94)	3.05 (1.68)	4.14 (4.28)	0.08	0.34	3.69 (2.63)	4.24 (4.17)	3.27 (2.51)	0.14	0.84
**QUICKI**	0.32 (0.03)	0.32 (0.02)	0.33 (0.04)	0.10	0.11	0.32 (0.03)	0.33 (0.03)	0.32 (0.03)	0.50	0.73	0.33 (0.04)	0.32 (0.03)	0.33 (0.03)	0.28	0.54

*SBP* Systolic Blood Pressure, *DBP* Diastolic Blood Pressure, *TC* Total Cholesterol, *TG* Triglyceride, *HDL-C* High Density Lipoprotein Cholesterol, *LDL-C* Low Density Lipoprotein Cholesterol, *HOMA-IR* Homeostatic Model Assessment for Insulin Resistance, *QUICKI* Quantitative Insulin sensitivity Check Index

^*^P values derived from One-Way ANOVA

^**^P values derived from ANCOVA after adjustment for confounders (age, gender, BMI, physical activity and energy intake)

**Table 3:** Biochemical variables of study participants by tertiles of high FODMAP

**Variable**	**Tertile of High FODMAP**				
	**1**^**st**^ ^**(*N*=112)**^	**2**^**nd**^ ^**(*N*=111)**^		**3**^**rd**^ ^**(*N*=112)**^	
		OR(CI)	*P*-value	OR(CI)	*P*-value
SBP (mmHg)					
Model I	**1 REF**	1.009 (0.993–1.025)	0.28	1.026 (1.008–1.044)	**0.004**
Model II		1.006 (0.989–1.023)	0.48	1.025 (1.006–1.044)	**0.009**
Model III		1.006 (0.978–1.035)	0.69	1.017 (0.982- 1.053)	0.33
DBP (mmHg)					
Model I	**1 REF**	1.006 (0.984–1.029)	0.57	1.025 (1.002–1.050)	**0.03**
Model II		1.003 (0.979–1.0262)	0.83	1.021 (0.996–1.047)	0.098
Model III		0.993 (0.959–1.028)	0.68	0.988 (0.946–1.032)	0.58
FBS (mg/dl)					
Model I	**1 REF**	1.003 (0.989–1.017)	0.70	1.003 (0.989–1.018)	0.64
Model II		1.001 (0.964–1.039)	0.96	1.004 (0.970–1.039)	0.82
Model III		1.005 (0.985–1.026)	0.61	1.015 (0.993–1.037)	0.17
TC (mg/dl)					
Model I	**1 REF**	1.019 (0.982–1.057)	0.32	1.002 (0.977–1.028)	0.85
Model II		1.021 (0.983–1.060)	0.29	1.004 (0.979–1.030)	0.75
Model III		0.999 (0.988–1.009)	0.78	0.996 (0.982–1.009)	0.50
TG (mg/dl)					
Model I	**1 REF**	0.995 (0.987–1.003)	0.18	0.998 (0.992–1.004)	0.52
Model II		0.994 (0.987–1.002)	0.16	0.998 (0.991–1.004)	0.47
Model III		0.999 (0.988–1.009)	0.78	0.996 (0.982–1.009)	0.50
HDL (mg/dl)					
Model I	**1 REF**	1.007 (0.980–1.035)	0.61	0.995 (0.967–1.023)	0.71
Model II		1.009 (0.980–1.037)	0.55	0.992 (0.964–1.021)	0.60
Model III		0.983 (0.943–1.024)	0.40	0.972 (0.923–1.024)	0.28
LDL (mg/dl)					
Model I	**1 REF**	1.006 (0.998–1.015)	0.14	1.003 (0.995–1.012)	0.46
Model II		1.006 (0.997–1.014)	0.17	1.002 (0.994–1.011)	0.58
Model III		1.000 (0.988–1.012)	0.99	0.995 (0.981–1.010)	0.51
Insulin (mIU/l)					
Model I	**1 REF**	1.000 (0.978–1.021)	0.96	0.994 (0.970–1.019)	0.64
Model II		0.998 (0.977–1.020)	0.86	0.990 (0.965–1.015)	0.42
Model III		1.041 (1.001–1.083)	**0.04**	1.010 (0.959–1.063)	0.71
HOMA-IR					
Model I	**1 REF**	1.010 (0.922–1.106)	0.83	0.993 (0.899–1.096)	0.88
Model II		1.003 (0.915–1.099)	0.95	0.973 (0.878–1.078)	0.59
Model III		1.159 (1.101-.1.351)	**0.049**	1.113 (0.919–1.348)	0.27
QUICKI					
Model I	**1 REF**	0.028 (3.890E-6–204.461)	0.43	3873.747 (0.002–6453005815)	0.25
Model II		0.032 (3.437E-6–296.630)	0.46	338.839 (0.077–1494435.114)	0.17
Model III		9.866E-7 (1.558E-12–0.625)	**0.04**	0.010 (3.245E-9–28934.052)	0.54

The multivariate multinomial logistic regression was used for estimation of ORs and confidence interval (CI). Model I: crude, Model II: adjusted for age and sex, Model III: adjusted for age, BMI, sex, SES, physical activity and energy intake

*SBP* Systolic Blood Pressure, *DBP* Diastolic Blood Pressure, *TC* Total Cholesterol, *TG* Triglyceride, *HDL-C* High Density Lipoprotein Cholesterol, *LDL-C* Low Density Lipoprotein Cholesterol, *HOMA-IR* Homeostatic Model Assessment for Insulin Resistance, *QUICKI* Quantitative Insulin sensitivity Check Index, *OR* Odds ratio, *CI* Confidence interval

**Table 4:** Biochemical variables of study participants by tertile of moderate FODMAP

**Variable**	**Tertile of Moderate FODMAP**				
	**1**^**st**^ ^**(*N*=112)**^	**2**^**nd**^ ^**(*N*=111)**^		**3**^**rd**^ ^**(*N*=112)**^	
		OR(CI)	*P*-value	OR(CI)	*P*-value
SBP (mmHg)					
Model I	**1 REF**	0.998 (0.982–1.014)	0.79	0.994 (0.978–1.010)	0.44
Model II		0.995 (0.978–1.012)	0.56	0.992 (0.975–1.010)	0.37
Model III		0.954 (0.919–0.991)	**0.01**	0.975 (0.940–1.012)	0.17
DBP (mmHg)					
Model I	**1 REF**	0.998 (0.976–1.021)	0.86	0.988 (0.966–1.011)	0.29
Model II		0.997 (0.973–1.021)	0.79	0.988 (0.965–1.012)	0.33
Model III		0.982 (0.942–1.024)	0.39	0.998 (0.957–1.040)	0.92
FBS (mg/dl)					
Model I	**1 REF**	0.987 (0.969–1.005)	0.16	1.008 (0.994–1.022)	0.26
Model II		0.987 (0.968–1.006)	0.16	1.008 (0.994–1.024)	0.26
Model III		0.987 (0.957–1.017)	0.37	1.007 (0.983–1.032)	0.55
TC (mg/dl)					
Model I	**1 REF**	0.999 (0.992–1.007)	0.86	1.000 (0.993–1.007)	0.96
Model II		1.000 (0.992–1.007)	0.90	1.000 (0.993–1.008)	0.93
Model III		0.999 (0.986–1.012)	0.87	1.009 (0.995–1.022)	0.19
TG (mg/dl)					
Model I	**1 REF**	1.003 (0.999–1.006)	0.10	1.002 (0.999–1.005)	0.22
Model II		1.002 (0.999–1.005)	0.19	1.001 (0.998–1.005)	0.35
Model III		1.000 (0.992–1.009)	0.94	1.007 (0.998–1.015)	0.11
HDL (mg/dl)					
Model I	**1 REF**	0.975 (0.948–1.003)	0.07	0 .990 (0.963–1.018)	0.47
Model II		0.980 (0.953–1.009)	0.17	0.996 (0.968–1.024)	0.77
Model III		0.949 (0.900–1.003)	0.051	0.982 (0.932–1.035)	0.50
LDL (mg/dl)					
Model I	**1 REF**	0.999 (0.991–1.007)	0.75	0.997 (0.989–1.006)	0.52
Model II		0.999 (0.991–1.007)	0.78	0.998 (0.989–1.006)	0.57
Model III		1.003 (0.989–1.017)	0.71	1.007 (0.9931.022)	0.33
Insulin (mIU/l)					
Model I	**1 REF**	0.976(0.946–1.007)	0.12	1.001 (0.981–1.021)	0.93
Model II		0.979 (0.949–1.010)	0.19	1.003 (0.983–1.024)	0.77
Model III		0.977 (0.928–1.028)	0.37	0.995(0.945–1.048)	0.84
HOMA-IR					
Model I	**1 REF**	0.872 (0.761–1.000)	0.05	1.018 (0.935–1.108)	0.68
Model II		0.885 (0.772–1.016)	0.08	1.030 (0.943 -1.125)	0.51
Model III		0.901 (0.735–1.104)	0.31	1.048 (0.871–1.260)	0.62
QUICKI					
Model I	**1 REF**	138.934 (0.026–751509.010)	0.26	3.388 (0.001–17835.160)	0.78
Model II		63.428 (0.011–367537.748)	0.34	1.649 (0.000–9463.304)	0.91
Model III		649.660 (0.000–3465157205.655)	0.41	1.091 (9.545E-8–12470267.859)	0.99

The multivariate multinomial logistic regression was used for estimation of ORs and confidence interval (CI). Model I: crude, Model II: adjusted for age and sex, Model III: adjusted for age, BMI, sex, SES, physical activity and energy intake

*SBP* Systolic Blood Pressure, *DBP* Diastolic Blood Pressure, *TC* Total Cholesterol, *TG* Triglyceride, *HDL-C* High Density Lipoprotein Cholesterol, *LDL-C* Low Density Lipoprotein Cholesterol, *HOMA-IR* Homeostatic Model Assessment for Insulin Resistance, *QUICKI* Quantitative Insulin sensitivity Check Index, *OR* Odds ratio, *CI* Confidence interval

**Table 5:** Biochemical variables of study participants by tertile of low FODMAP

**Variable**	**Tertile of Low FODMAP**				
	**1**^**st**^ ^**(*N*=112)**^	**2**^**nd**^ ^**(*N*=111)**^		**3**^**rd**^ ^**(*N*=112)**^	
		OR(CI)	*P*-value	OR(CI)	*P*-value
SBP (mmHg)					
Model I	**1 REF**	1.008 (0.992–1.025)	0.30	1.004 (0.989–1.021)	0.58
Model II		1.005 (0.988–1.023)	0.58	0.999 (0.982–1.016)	0.92
Model III		1.007 (0.976–1.039)	0.67	0.990 (0.956–1.026)	0.59
DBP (mmHg)					
Model I	**1 REF**	1.020 (0.997–1.043)	0.09	1.002 (0.980–1.025)	0.83
Model II		1.017 (0.993–1.042)	0.17	0.998 (0.975–1.022)	0.86
Model III		1.031 (0.992–1.072)	0.12	1.027 (0.984–1.071)	0.22
FBS (mg/dl)					
Model I	**1 REF**	1.006 (0.990–1.022)	0.46	1.009 (0.994–1.025)	0.23
Model II		1.005 (0.989–1.022)	0.54	1.008 (0.992–1.024)	0.31
Model III		1.006 (0.979–1.033)	0.66	1.009 (0.981–1.037)	0.54
TC (mg/dl)					
Model I	**1 REF**	1.000 (0.993–1.007)	0.98	0.997 (0.990–1.004)	0.42
Model II		1.015 (0.985–1.045)	0.335	0.996 (0.989–1.004)	0.33
Model III		1.006 (0.993–1.018)	0.38	1.004 (0.991–1.018)	0.51
TG (mg/dl)					
Model I	**1 REF**	1.002 (0.999–1.005)	0.19	0.999 (0.996–1.002)	0.44
Model II		1.000 (0.997–1.004)	0.32	0.998 (0.994–1.001)	0.16
Model III		1.007 (0.999–1.015)	0.10	1.004 (0.996–1.013)	0.30
HDL (mg/dl)					
Model I	**1 REF**	0.997 (0.970–1.025)	0.82	0.997 (0.970–1.025)	0.83
Model II		1.000 (0.972–1.028)	0.97	1.004 (0.976–1.033)	0.76
Model III		0.990 (0.945–1.036)	0.65	0.999 (0.950–1.050)	0.95
LDL (mg/dl)					
Model I	**1 REF**	0.990 (0.954–1.028)	0.60	1.002 (0.976–1.030)	0.85
Model II		0.997 (0.989–1.005)	0.51	0.993 (0.985–1.002)	0.11
Model III		1.003 (0.990–1.017)	0.62	1.003 (0.989–1.018)	0.68
Insulin (mIU/l)					
Model I	**1 REF**	1.011 (0.988–1.035)	0.34	0.975 (0.944–1.007)	0.12
Model II		1.011 (0.987–1.037)	0.37	0.978 (0.946–1.011)	0.19
Model III		0.986 (0.942–1.032)	0.55	0.959 (0.910–1.011)	0.12
HOMA-IR					
Model I	**1 REF**	1.047 (0.953–1.151)	0.33	0.941 (0.834–1.062)	0.32
Model II		1.047 (0.947–1.157)	0.36	0.950 (0.840–1.075)	0.41
Model III		0.956 (0.809–1.129)	0.59	0.918 (0.758–1.112)	0.38
QUICKI					
Model I	**1 REF**	0.007 (8.253E-7–52.461)	0.27	6.835 (.002–28120.029)	0.65
Model II		0.005 (5.607E-7–42.133)	0.25	3.065 (.001–16848.704)	0.79
Model III		0.030 (1.213E-8–73209.130)	0.64	26.691 (6.507E-6–109485962.649)	0.67

The multivariate multinomial logistic regression was used for estimation of ORs and confidence interval (CI). Model I: crude, Model II: adjusted for age and sex, Model III: adjusted for age, BMI, sex, SES, physical activity and energy intake

*SBP* Systolic Blood Pressure, *DBP* Diastolic Blood Pressure, *TC* Total Cholesterol, *TG* Triglyceride, *HDL-C* High Density Lipoprotein Cholesterol, *LDL-C* Low Density Lipoprotein Cholesterol, *HOMA-IR* Homeostatic Model Assessment for Insulin Resistance, *QUICKI* Quantitative Insulin sensitivity Check Index, *OR* Odds ratio, *CI* Confidence interval

**Table 6:** Dietary intake of participants across different tertiels of High FODMAP, Moderate FODMAP and Low FODMAP intake

**Variables**	**High FODMAP** **(**0.02–1.22g/ kcal)				**Moderate FODMAP** **(**0.03–0.39 g/ kcal**)**				**Low FODMAP** **(**0.13–2.96 g/ kcal**)**			
	T1 (*n* = 113)	T2 (*n* = 113)	T3 (*n* = 113)	*P	T1 (*n* = 113)	T2 (*n* = 113)	T3 (*n* = 113)	*P	T1 (*n* = 113)	T2 (*n* = 113)	T3 (*n* = 113)	*P
**Energy (kcal/day)**	2394.15 (752.22)	2965.64 (922.67)	3680.23 (1172.71)	< 0.001	2300.23 (751.17)	3008.89 (916.42)	3746.94 (1083.18)	< 0.001	2410.52 (729.82)	2965.82 (908.69)	3685.71 (1208.14)	< 0.001
**Protein (g/day)**	77.01 (22.75)	98.46 (28.21)	123.49 (41.96)	< 0.001	75.84 (22.20)	102.32 (36.55)	121.08 (35.92)	< 0.001	82.64 (25.90)	98.63 (31.59)	118.12 (43.16)	< 0.001
**Fat (g/day)**	81.60 (36.58)	98.72 (42.19)	120.20 (52.59)	< 0.001	79.53 (41.09)	99.72 (41.94)	122.27 (48.30)	< 0.001	81.57 (34.49)	99.72 (44.10)	120.41 (53.10)	< 0.001
**CHO (g/day)**	352.55 (119.67)	442.29 (136.67)	559.35 (180.87)	< 0.001	338.42 (119.25)	449.61 (132.09)	567.67 (170.38)	< 0.001	355.19 (109.01)	441.69 (141.90)	559.79 (184.85)	< 0.001
**Total Fiber (g/day)**	55.08 (30.99)	68.30 (41.05)	74.55 (40.66)	0.01	40.39 (13.18)	59.03 (19.01)	98.65 (46.40)	< 0.001	47.78 (20.13)	62.05 (29.38)	88.78 (48.74)	< 0.001
**SFA (g/day)**	21.71 (9.01)	27.98 (10.29)	38.16 (18.72)	< 0.001	23.56 (12.93)	29.78 (13.37)	34.65 (16.39)	< 0.001	24.61 (11.45)	28.93 (14.26)	34.49 (17.14)	< 0.001
**MUFA (g/day)**	27.91 (14.18)	32.71 (16.23)	38.77 (17.40)	< 0.001	26.68 (14.78)	32.70 (14.34)	40.44 (17.92)	< 0.001	27.35 (12.20)	32.67 (16.00)	39.86 (18.93)	< 0.001
**PUFA(g/day)**	20.18 (11.49)	22.59 (13.20)	24.64 (14.10)	0.37	17.55 (10.62)	22.20 (12.49)	28.05 (14.31)	< 0.001	17.88 (8.54)	21.97 (12.17)	28.01 (16.04)	< 0.001
**Cholesterol (mg/day)**	262.46 (260.87)	286.28 (146.88)	342.17 (165.04)	0.08	262.14 (250.77)	291.23 (152.06)	336.79 (175.81)	0.01	256.15 (248.76)	295.91 (153.92)	338.51 (175.69)	0.08
**Sodium (mg/day)**	4018.01 (1494.22)	4948.49 (2501.65)	5093.06 (2435.91)	< 0.001	3392.70 (1425.93)	4692.32 (1891.29)	5980.32 (2460.93)	< 0.001	3817.03 (1582.76)	4462.57 (1715.54)	5805.45 (2759.34)	< 0.001
**Iron (mg/day)**	20.21 (11.69)	23.64 (8.43)	27.45 (10.74)	< 0.001	16.61 (4.98)	23.51 (10.87)	31.28 (9.91)	< 0.001	18.39 (5.34)	23.83 (11.77)	29.28 (11.11)	< 0.001
**Magnesium (mg/day)**	414.79 (153.80)	531.24 (161.59)	683.27 (275.09)	< 0.001	393.90 (132.45)	561.92 (235.92)	675.29 (219.48)	< 0.001	424.36 (142.20)	524.93 (156.10)	683.99 (288.95)	< 0.001
**Zinc (mg/day)**	11.27 (3.97)	14.53 (4.30)	18.59 (9.04)	< 0.001	10.86 (3.62)	15.51 (7.94)	18.04 (6.33)	< 0.001	11.99 (3.84)	14.32 (4.72)	18.14 (9.33)	< 0.001
**Phosphorus (mg/day)**	1329.29 (388.43)	1780.02 (477.14)	2307.49 (682.05)	< 0.001	1357.86 (420.84)	1864.23 (627.61)	2195.72 (626.44)	< 0.001	1492.36 (490.30)	1780.78 (558.87)	2149.71 (745.55)	< 0.001
**Calcium (mg/day)**	863.28 (276.11)	1245.52 (361.30)	1761.90 (599.67)	< 0.001	963.32 (399.33)	1316.35 (567.03)	1591.79 (540.91)	< 0.001	1042.95 (425.20)	1272.72 (488.78)	1560.10 (649.40)	< 0.001
**Potassium (mg/day)**	3205.38 (1022.82)	4504.79 (1095.49)	6534.92 (2182.49)	< 0.001	3753.55 (1636.09)	4894.63 (1938.79)	5586.84 (1221.20)	< 0.001	3763.87 (1570.68)	4570.17 (1575.04)	5919.11 (2318.83)	< 0.001
**Copper (mg/day)**	1.97 (0.93)	2.46 (1.00)	2.98 (1.27)	0.008	1.75 (0.71)	2.45 (1.01)	3.22 (1.17)	< 0.001	1.91 (0.62)	2.41 (0.88)	3.12 (1.46)	< 0.001
**Manganese (mg/day)**	8.04 (3.94)	9.17 (3.50)	9.58 (3.89)	< 0.001	6.19 (2.17)	9.09 (3.11)	11.54 (3.90)	< 0.001	6.28 (2.19)	8.50 (2.48)	12.11 (4.00)	< 0.001
**Selenium (mg/day)**	133.37 (57.09)	157.12 (59.23)	166.01 (65.69)	< 0.001	105.58 (38.08)	150.50 (39.92)	200.83 (63.08)	< 0.001	124.31 (46.65)	148.40 (55.92)	184.81 (67.08)	< 0.001
**Fluorine (mg/day)**	3478.65 (3660.77)	3351.47 (2421.67)	3563.03 (2982.08)	0.78	2861.04 (2406.17)	3618.24 (3817.32)	3905.10 (2699.92)	0.02	1408.95 (793.45)	2894.18 (1061.32)	6125.63 (3857.18)	< 0.001
**Chromium (mg/day)**	0.13 (0.11)	0.17 (0.12)	0.16 (0.11)	0.05	0.08 (0.05)	0.16 (0.08)	0.22 (0.14)	< 0.001	0.12 (0.09)	0.15 (0.10)	0.19 (0.14)	< 0.001
Vitamin C (mg/day)	126.97 (89.40)	217.94 (117.76)	374.54 (208.92)	< 0.001	198.48 (173.16)	244.51 (159.96)	276.30 (195.16)	0.04	192.90 (144.29)	238.48 (157.80)	288.69 (216.38)	< 0.001
VitaminB1 (mg/day)	2.24 (0.84)	2.65 (1.04)	2.96 (1.16)	< 0.001	1.78 (0.46)	2.55 (0.66)	3.53 (1.09)	< 0.001	2.04 (0.62)	2.55 (0.87)	3.28 (1.21)	< 0.001
VitaminB2 (mg/day)	1.89 (0.75)	2.51 (0.76)	3.27 (1.06)	< 0.001	1.89 (0.67)	2.60 (0.93)	3.19 (1.02)	< 0.001	2.05 (0.72)	2.52 (0.93)	3.12 (1.12)	< 0.001
VitaminB3 (mg/day)	24.93 (8.35)	29.89 (10.70)	33.66 (12.14)	< 0.001	21.20 (5.78)	29.35 (7.62)	38.03 (11.61)	< 0.001	23.78 (7.21)	28.99 (9.29)	35.92 (12.59)	< 0.001
VitaminB6 (mg/day)	1.77 (0.72)	2.24 (0.61)	2.97 (0.91)	< 0.001	1.76 (0.58)	2.43 (0.89)	2.80 (0.88)	< 0.001	1.88 (0.64)	2.33 (0.81)	2.78 (1.00)	< 0.001
VitaminB9 (μg/day)	608.40 (213.10)	723.97 (287.16)	830.78 (323.58)	< 0.001	530.98 (147.63)	689.68 (195.77)	944.26 (329.74)	< 0.001	4.18 (2.96)	5.18 (6.07)	6.68 (7.71)	< 0.001
VitaminB12 (μg/day)	4.19 (7.03)	5.20 (5.45)	6.64 (5.07)	< 0.001	4.20 (4.74)	5.88 (7.32)	5.94 (5.47)	0.04	5.80 (1.95)	6.81 (2.19)	8.04 (2.70)	0.07
VitaminB5 (mg/day)	4.97 (1.42)	6.68 (1.52)	8.97 (2.46)	< 0.001	5.53 (1.93)	7.12 (2.30)	7.98 (2.50)	< 0.001	5.80 (1.95)	6.81 (2.19)	8.04 (2.70)	< 0.001
VitaminB8 (mg/day)	29.47 (12.04)	40.92 (13.50)	54.35 (19.32)	< 0.001	31.03 (11.81)	42.17 (15.34)	51.44 (20.60)	< 0.001	35.13 (16.55)	40.45 (14.30)	49.17 (20.85)	< 0.001
Vitamin A (RAE/day)	665.47 (707.01)	830.55 (545.97)	1204 (682.61)	< 0.001	712.79 (544.46)	927.92 (745.26)	1065.15 (708.63)	< 0.001	727.32 (461.78)	874.49 (658.81)	1107.22 (836.46)	< 0.001
Vitamin D (μg/day)	1.27 (0.80)	1.92 (1.26)	2.92 (1.79)	< 0.001	1.85 (1.36)	2.14 (1.57)	2.03 (1.50)	0.29	1.87 (1.45)	2.07 (1.53)	2.15 (1.53)	0.34
Vitamin K (μg/day)	185.36 (155.17)	224.39 (153.78)	336.66 (323.78)	< 0.001	200.88 (162.96)	228.40 (167.97)	328.50 (340.42)	< 0.001	199.73 (168.96)	257.46 (215.20)	301.90 (317.70)	0.07
Vitamin E (mg/day)	13.98 (7.98)	16.16 (8.38)	18.26 (7.64)	< 0.001	13.33 (7.78)	15.63 (7.02)	19.71 (8.80)	< 0.001	13.21 (6.60)	16.25 (8.79)	19.28 (8.32)	< 0.001

All data are mean (± SD)

*CHO* Carbohydrate, *SFA* Saturated fatty acids, *MUFA* Mono-unsaturated fatty acids, *PUFA* Polyunsaturated fatty acids

^*^P values derived from One-Way ANOVA

**Table 7:** Food groups intake of study participants across different tertiels of High FODMAP, Moderate FODMAP and Low FODMAP intake

**Variables**	**High FODMAP** **(**0.02–1.22 g/ kcal)				**Moderate FODMAP** **(**0.03–0.39 g/ kcal**)**				**Low FODMAP** **(**0.13–2.96 g/ kcal**)**			
	T1 (*n* = 113)	T2 (*n* = 113)	T3 (*n* = 113)	*P	T1 (*n* = 113)	T2 (*n* = 113)	T3 (*n* = 113)	*P	T1 (*n* = 113)	T2 (*n* = 113)	T3 (*n* = 113)	*P
**Fruits (g/d)**	2.90 (3.18)	4.28 (2.45)	6.30 (2.70)	< 0.001	3.46 (2.27)	4.77 (4.11)	4.36 (2.89)	0.06	3.30 (1.99)	4.34 (3.32)	4.76 (3.58)	0.02
**Vegetables (g/d)**	2.95 (1.54)	3.65 (1.46)	5.75 (3.09)	< 0.001	1.58 (0.19)	3.64 (1.57)	4.78 (2.81)	< 0.001	3.06 (1.81)	3.73 (1.55)	4.67 (2.83)	< 0.001
**MFP (g/d)**	2.78 (1.53)	3.22 (1.70)	4.13 (1.93)	< 0.001	2.69 (1.21)	3.60 (1.95)	3.51 (1.91)	< 0.001	2.84 (1.49)	3.24 (1.64)	3.61 (1.99)	0.04
**Dairy (g/d)**	1.13 (0.48)	2.14 (0.80)	3.70 (1.29)	< 0.001	1.89 (1.13)	2.27 (1.56)	2.10 (1.25)	0.30	1.88 (1.40)	2.12 (1.20)	2.20 (1.31)	0.36
**Grains (g/d)**	13.14 (5.78)	15.37 (7.18)	14.46 (8.22)	0.15	8.32 (2.90)	13.90 (3.67)	19.88 (6.75)	< 0.001	10.62 (4.34)	13.67 (6.22)	17.99 (7.60)	< 0.001
**Nuts(g/day)**	11.63 (20.13)	14.33 (15.06)	25.94 (65.62)	0.02	11.94 (19.98)	21.73 (55.33)	18.10 (38.97)	0.19	11.52 (14.12)	15.94 (21.72)	24.36 (65.53)	0.049
**Beans(g/day)**	0.55 (0.39)	0.87 (0.89)	0.89 (0.78)	< 0.001	0.63 (0.55)	0.70 (0.58)	0.87 (0.87)	0.12	0.62 (0.52)	0.69 (0.40)	0.89 (0.98)	0.09
**Fiber (g/day)**	61.10 (33.26)	78.37 (45.27)	82.37 (54.80)	0.01	38.97 (10.94)	65.25 (19.86)	107.00 (49.39)	< 0.001	50.63 (23.14)	67.45 (34.45)	95.29 (53.92)	< 0.001

All data are mean (± SD)

*MFP* Meat, fish and poultry

^*^P values derived from One-Way ANOVA

## Discussion

To our knowledge, this research was the first to examine the relationship between high, moderate, and low FODMAP diets and MetS risk factors among people with obesity in Tabriz and Tehran, Iran. Before this study, another similar study was conducted at the Prince of Wales Hospital (PWH) of the Chinese University of Hong Kong on individuals with impaired glucose tolerance (IGT) [10]. This study found that participants with IGT had the lowest daily FODMAP intake compared to their non-overweight and non-obese counterparts, despite having similar total daily energy intake. The total content of FODMAPs was negatively correlated with body fat [10]. This study found that higher consumption of moderate FODMAP and low FODMAP groups was associated with lower WHR and higher FFM. To explain this association, we have proposed the following hypothesis: a moderate increase in FODMAP consumption was linked to an absolute rise and a relative abundance of microbiota that produce SCFAs [41–43]. Peroxisome proliferator-activated receptor-γ (PPAR-γ) is a transcription factor whose activity can be modulated by SCFA in this. In addition to reducing ectopic fat buildup and improving lipid and glucose metabolism, the latter may also control adipocyte differentiation [44, 45].

Additionally, these bacterial metabolites can stimulate the sympathetic nervous system and restore the activity of gastrointestinal and endocrine cells by increasing the secretion of gut hormones like PYY, GLP-1, and cholecystokinin (CKK). Examples of these metabolites include Akkermansia muciniphila phospholipids. Gluconeogenesis, glycogenolysis, and lipolysis from adipose tissues can all be systemically regulated by these changes in the metabolic and hormonal milieu [46, 47]. Additionally, these hormones may act on the brain-gut axis to control food consumption by enhancing epigastric fullness and satiety [48, 49]. Animal [50, 51] and human trials [52, 53] revealed an inverse relationship between body fat content and Akkermansia muciniphila. From these explanations, high amounts of low and moderate FODMAP foods may be associated with better lipid and glucose metabolism and lower food intake. Therefore, individuals with this condition may have a higher FFM and a lower WHR.

The relationship between high amounts of high FODMAP intake and blood pressure did not remain significant after adjusting for age, sex, SES, energy intake, and physical activity. It is shown that with aging [54] and increasing BMI [55], SBP rises. According to the study of Moghaddam et al. [56], the association between dietary patterns and SBP became non-significant after adjusting for age, sex, marital, smoking, income, body mass index, waist-to-hip ratio, family history of hypertension, energy intake and physical activity level. Another interesting finding of this study was that consuming moderate FODMAP foods was associated with lower SBP, a significant in the second tertile. It is proven that moderate and low FODMAP foods contain high amounts of potassium [57]. In our research of scientific resources, we found that potassium alters the neural mechanisms in either the central or peripheral systems that control blood pressure.

Moreover, potassium-rich diets might lower blood pressure by inducing relaxation in the smooth muscle of blood vessels and directly decreasing resistance in the peripheral vasculature [58]. We showed that higher serum insulin levels, HOMA-IR, and lower QUICKI were observed in individuals consuming higher amounts of high FODMAP foods. Lower values of QUICKI may indicate greater insulin resistance [59], and high HOMA-IR values indicate low insulin sensitivity [60]. In the literature, we found different and even contradictory results. As mentioned, high-FODMAP foods contain high amounts of fructose [61]. Exposure of the liver to high fructose levels induces lipogenesis and TG buildup acceleration, which reduces insulin sensitivity and increases hepatic insulin resistance and glucose intolerance [62].

**Fig. 1:**
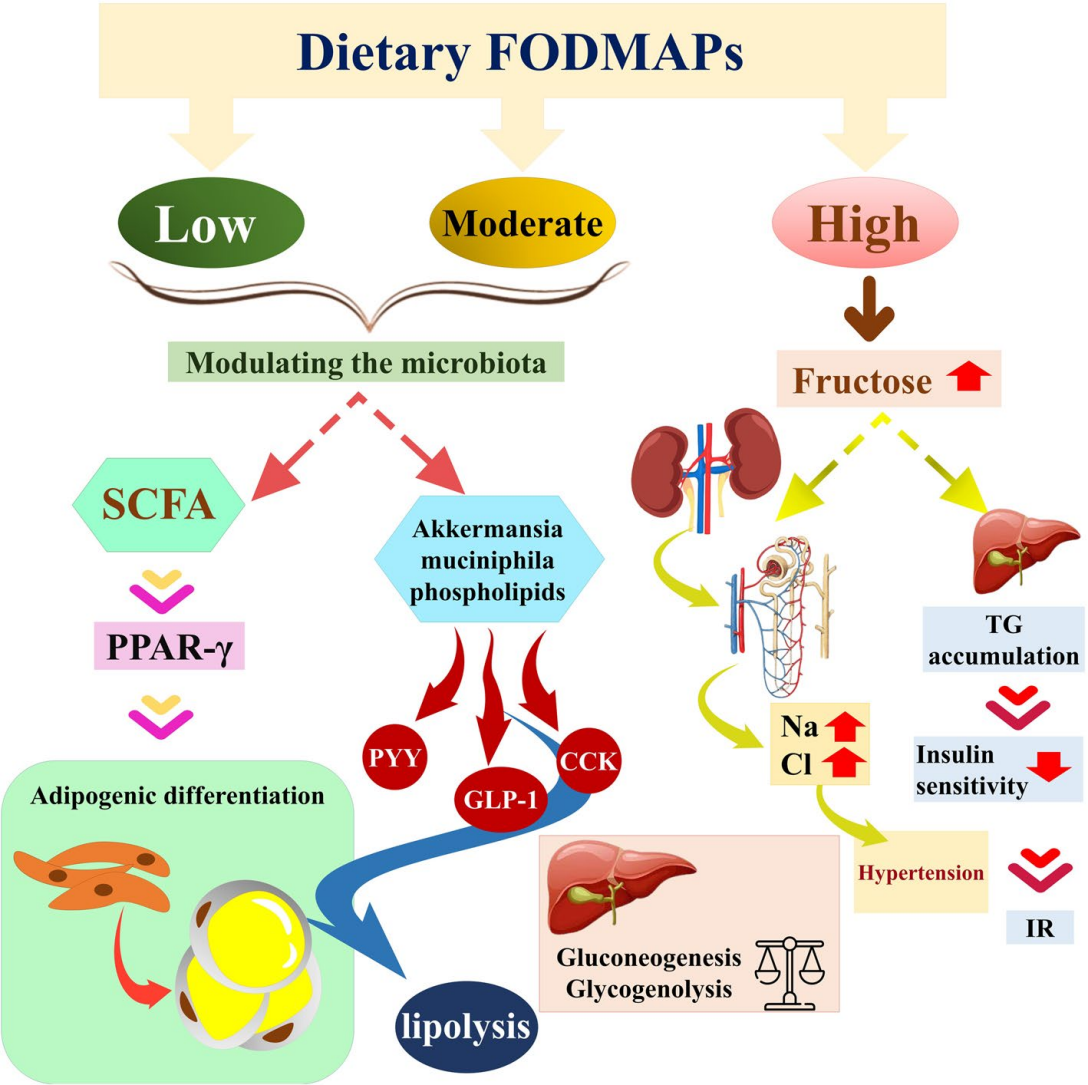
Graphical abstract of consuming different amounts of FODMAPs on cardiometabolic factors. Consumption of low and medium amounts of FODAMPs can work through the effect on the microbial population and their metabolites in lipid and glucose metabolism, as well as adipogenic differentiation. While high FODMAPs in the diet due to the high amount of fructose with the effect of increasing the absorption of sodium and chloride and reducing their excretion causes an increase in blood pressure, also when the liver is exposed to high fructose, the accumulation of triglycerides causes a decrease in insulin sensitivity and then insulin resistance. FODMAP, fermentable oligosaccharides, disaccharides, monosaccharides and polyols; SCFA, Short-chain fatty acids; PPAR-γ, peroxisome proliferator-activated receptor-γ, PYY, peptide YY; GLP-1, glucagon-like peptide 1; CCL, cholecystokinin; TG, triglyceride, IR, insulin resistance

In contrast, a study showed that, compared to a low FODMAP diet, a 24-h high FODMAP diet in healthy subjects decreased the amount of lipopolysaccharide (LPS) binding protein. It is known that LPS and persistent subclinical inflammation both raise insulin intolerance [63, 64]. The summarized mechanistic pathways of the possible health effects of dietary FODMAP are represented in Fig. 1.

### Limitation

This study was a cross-sectional study to assess the association between FODMAP diet and metabolic syndrome. Several limitations of this study should be addressed; first of all, the causality inference is impossible due to the study’s observational design. Therefore, performing clinical trials is suggested which reduces insulin sensitivity and increase dress this issue. Also, using FFQ as a subjective tool to collect dietary information is a matter of recall bias. However, it should be noted that our semi-quantitative FFQ was a valid and reliable tool adopted by the Iranian population.

In conclusion, this study evaluated the association of foods with different amounts of FODMAP and metabolic syndrome in the Iranian population. The findings of this study revealed that consuming low and moderate FODMAP foods is associated with lower WHR, higher FFM, and SBP. Conversely, consuming higher amounts of high-FODMAP foods is associated with insulin resistance. It can be suggested that high-FODMAP foods might be harmful to people with metabolic syndrome. These findings highlight the potential role of FODMAP in managing metabolic syndrome and open a new field of research.

## Data Availability

The datasets generated and/or analyzed during the current study are not publicly available due to privacy and ethical considerations, but can be obtained from the corresponding author on reasonable request.
